# AFF1 inhibits adipogenic differentiation via targeting TGM2 transcription

**DOI:** 10.1111/cpr.12831

**Published:** 2020-05-22

**Authors:** Yaqian Chen, Yuan Wang, Weimin Lin, Rui Sheng, Yunshu Wu, Ruoshi Xu, Chenchen Zhou, Quan Yuan

**Affiliations:** ^1^ State Key Laboratory of Oral Diseases National Clinical Research Center for Oral Diseases West China Hospital of Stomatology Sichuan University Chengdu China

**Keywords:** 3T3‐L1 pre‐adipocytes, adipogenic differentiation, AF4/FMR2 family member 1, human mesenchymal stromal/stem cells, TGM2

## Abstract

**Objectives:**

AF4/FMR2 family member 1 (AFF1), known as a central scaffolding protein of super elongation complex (SEC), regulates gene transcription. We previously reported that AFF1 inhibited osteogenic differentiation of human mesenchymal stromal/stem cells (hMSCs). However, its role in adipogenic differentiation has not been elucidated.

**Materials and methods:**

hMSCs and 3T3‐L1 pre‐adipocytes were cultured and induced for adipogenic differentiation. Small interfering RNAs (siRNAs) were applied to deplete *AFF1* while lentiviruses expressing HA*‐*Aff1 were used for overexpression. Oil Red O staining, triglyceride (TAG) quantification, quantitative real‐time PCR (qPCR), Western blot analysis, immunofluorescence staining, RNA sequencing (RNA‐seq) analysis and ChIP‐qPCR were performed. To evaluate the adipogenesis in vivo, BALB/c nude mice were subcutaneously injected with *Aff1*‐overexpressed 3T3‐L1 pre‐adipocytes.

**Results:**

AFF1 depletion leads to an enhanced adipogenesis in both hMSCs and 3T3‐L1 pre‐adipocytes. Overexpression of Aff1 in 3T3‐L1 cells results in the reduction of adipogenic differentiation and less adipose tissue formation in vivo. Mechanistically, AFF1 binds to the promoter region of *Tgm2* gene and regulates its transcription. Overexpression of Tgm2 largely rescues adipogenic differentiation of *Aff1‐*deficient cells.

**Conclusions:**

Our data indicate that AFF1 inhibits adipogenic differentiation by regulating the transcription of TGM2.

## 1 INTRODUCTION

Adipose tissues, composed with mature adipocytes and pre‐adipocytic stem cells, play crucial roles in maintaining whole‐body health and energy metabolism.^1^ Mature adipocytes are derived and differentiated from mesenchymal stromal/stem cells (MSCs) or pre‐adipocytes. The process of cellular adipogenic differentiation, so‐called adipogenesis, is regulated by various signalling pathways and transcription factors.^2^, ^3^ Adipogenesis is a sequentially orchestrated process involving the expression of multiple vital factors, among which CCAAT/enhancer‐binding protein‐β (C/EBPβ) is a well‐studied early adipogenic transcription factor that, in turn, induces the expression of two master adipogenic transcription factors, peroxisome proliferator‐activated receptor‐γ (PPARγ) and C/EBPα.^4^, ^5^ PPARγ and C/EBPα then work in cooperation to activate many adipocyte‐specific genes (*PEPCK*, *aP2* and *GLUT4*),^6^ thus initiating the adipogenic cascade.^7^

Recently, epigenomic factors, involving histone modifications, DNA methylation and chromatin remodelling have been reported to regulate adipogenesis.^8^, ^9^, ^10^ Another important transcriptional regulation is transcription elongation of related adipogenic genes. The elongation stage of RNA polymerase (Pol) II transcription plays an indispensable role in controlling the gene transcription level of many cells and viruses.^11^ For instance, ATF4 has been identified as a positive transcription regulator of adipogenesis in hMSCs as it forms heterodimers with enhancers, facilitating RNA Pol II binding to hybrid motifs of C/EBPs and thus continuing relative gene transcription.^12^

Super elongation complex (SEC), containing positive transcription elongation factor b (P‐TEFb), is a major transcription elongation regulator of RNA Pol II.^13^ Within the SEC, the AF4*/*FMR2 family proteins AFF1 and AFF4 perform as a central scaffold which consolidates the flexible complex with short hydrophobic regions and interacts with other subunits of SEC.^14^ AFF1 functions as a positive regulator of P‐TEFb kinase, regulating the transcription of a large number of genes.^15^, ^16^ Furthermore, AFF1 has been recognized as a related factor of systemic diseases, such as acute lymphoblastic leukaemia and FRAXE mental retardation.^17^, ^18^ AFF1 also plays a significant role in HIV transactivation and is highly associated with HIV‐1 Tat.^19^, ^20^

Our previous study demonstrated AFF1 suppressed cellular osteogenic differentiation in vitro and decreased ectopic bone formation in vivo by controlling the transcription of DDK1.^21^ However, its effect on adipogenic differentiation is unclear. The aim of the present study is to unveil the possible impact and mechanism of AFF1 on mesenchymal stem cell adipogenic differentiation and pre‐adipocyte maturation.

## 2 MATERIALS AND METHODS

### 2.1 Cell culture and differentiation

Human MSCs and 3T3‐L1 pre‐adipocytes were obtained from American Type Culture Collection (ATCC). Cells were cultured as previously described.^22^ Briefly, cells were cultured in Minimum Essential Medium Eagle – Alpha Modification (alpha‐MEM; HyClone) or Dulbecco's Modified Eagle Medium (DMEM; HyClone) supplemented with 10% foetal bovine serum (FBS; Gibco), 100 units mL^−1^ penicillin/100 µg mL^−1^ streptomycin (Gibco). Cells were grown on 100 cm^2^ dish (Corning) at 37°C in an atmosphere of 5% CO_2_ and were passaged at 90% confluence.

To induce adipogenic differentiation, cells were seeded in culture plates and treated with adipogenic medium consisting of 0.5 μM isobutylmethylxanthine, 10 μg/mL insulin, and 1 μM dexamethasone (all from Sigma). All experimental protocols and procedures were approved by the State Key Laboratory of Oral Diseases, West China Hospital of Stomatology, Sichuan University.

### 2.2 Gene knockdown and overexpression

For gene knockdown, targeted small interfering RNAs (siRNAs) were purchased from Santa Cruz Biotechnology (sc‐60131, sc‐60132). Control siRNAs were designed and obtained from Sangon Biotech. Transfections were performed using Lipofectamine RNAiMAX reagent (Invitrogen) according to the manufacturer's instructions. After transfection for 2 days, the knockdown efficiency was confirmed by quantitative real‐time PCR (qPCR) and Western blot.

For gene overexpression, lentiviruses expressing HA‐Aff1 or empty vectors were designed and purchased from GeneCopoeia. Cells were infected with viruses in the presence of polybrene (Sigma) for 24 hours and selected by 1 μg mL^−1^ puromycin addition (Sigma). The infection efficiency of overexpression was verified by qPCR and Western blot.

### 2.3 Oil Red O staining and quantification

Oil Red O (ORO) staining was used to measure endocellular lipid accumulation. As previously described,^23^ cells were fixed in 4% paraformaldehyde for 20 minutes after 1‐2 weeks of adipogenic induction. After washed with PBS twice, cells were stained with 0.5% ORO (Sigma) in 60% isopropanol for 30 minutes at room temperature. Cells were washed with PBS for another three times to remove the non‐specific staining.

The incorporated staining was then dissolved in isopropanol and transferred into a 96‐well plate. Optical density (OD) values of absorbance at 500 nm were measured by a spectrophotometer (Thermo Fisher Scientific). Relative values were calculated comparing experimental samples to controls.

### 2.4 Triglyceride (TAG) quantification

After 1‐2 weeks of adipogenic induction, cells were trypsinized and centrifuged (250 *g*, 5 minutes). We washed cells with cold PBS twice, resuspended and homogenized samples in 0.5 mL of 5% NP‐40 solution. Then, slowly heated the samples to 80‐100°C until the NP‐40 solution became cloudy and then cooled down to room temperature. This step was repeated twice to solubilize all triglycerides. To remove insoluble material, cells were centrifuged at top speed in a microcentrifuge for 2 minutes The TAG content of cell sample was detected using a commercial kit (Abcam, ab65336) following manufacturer's instructions. OD values were detected at 570 nm, and TAG concentrations were calculated according to the standard curve.

### 2.5 Quantitative real‐time PCR (qPCR)

Total RNA of the cells was isolated with TRIzol reagent (Invitrogen) following manufacturer's instructions.^24^ Reverse transcription was performed using PrimeScript RT Reagent kit with gDNA Eraser (Takara), and then, cDNA was prepared from 1μg RNA. qPCR was accomplished applying SYBR Premix Ex Taq II (Takara) in Bio‐Rad CFX96 Real‐Time System. The relative mRNA expressions of corresponding genes were normalized by housekeeping genes and calculated using the 2^−∆∆^Ct method, comparing experimental samples to controls. The primers are listed in Table S1.

### 2.6 Adipose formation model

3T3‐L1 pre‐adipocytes were collected, washed and resuspended in cold PBS. We subcutaneously injected 10^7^ cells (500 μL) in 50% Matrigel (cat.356234, Becton Dickinson) suspension into the sternal area of athymic BALB/c nude mice. After 6 weeks of implantation, mice were anesthetized and the fat pads were excised. After carefully removing the adjacent skin and subcutaneous muscle tissues, the transplants were weighed. Then, the engrafted tissues were fixed in 4% paraformaldehyde overnight and proceeded to histologically analyses as previously described.^25^ The number of adipocytes (/mm^2^) and the percentage of fat area versus total tissue area (%) were calculated via ImageJ software. Statistics were calculated in three replicate views of each sample.

### 2.7 Western blot

Cells were lysed in RIPA buffer (Pierce) on ice as previously described and centrifuged at 15 000 *g* for 15 minutes at 4°C to remove the cell debris.^26^ The supernatants were heated at 95°C for 5 minutes in sample buffer containing 2% SDS and 1% 2‐mercaptoethanol, separated on SDS‐polyacrylamide gels and transferred to PVDF membranes using a wet transfer apparatus (Bio‐Rad). The membranes were blocked with 5% BSA in PBS for 1 hour at room temperature (RT), then incubated overnight at 4°C with primary antibodies. Primary antibodies used in this study were as follows: rabbit anti‐α tubulin (#11224‐1‐AP, Proteintech, 1:2000); rabbit anti‐AFF1 (#A302‐344A, Bethyl, 1:1000); and rabbit anti‐TGM2 (#15100‐1‐AP, Proteintech, 1:2000). The next day, blots were incubated with HRP‐conjugated secondary antibodies (#L3012, SAB, 1:5000) at RT for 1 hour and antibody‐antigen complexes were visualized and detected with Immobilon reagents (Millipore).

### 2.8 Immunofluorescence staining

Cells were cultured on clean glass slides in 24‐well plates. Upon harvest, cells were washed with pre‐cooled PBS twice and fixed with 4% paraformaldehyde at RT for 20 minutes. To block non‐specific staining, cells were incubated with 4% BSA in PBS for 30 minutes at 37°C. Primary antibodies were then applied to wells, interacting with cells at 4°C overnight. Primary antibodies used in this study were as follows: rabbit anti‐AFF1 (#A302‐344A, Bethyl, 1:200); and rabbit anti‐TGM2 (#15100‐1‐AP, Proteinch, 1:100). The next day, cells were rinsed with PBS twice for 5 minutes and incubated with the corresponding secondary antibody (Jackson Immuno, 1:200) at RT for 1 hour. Cells were washed with PBS for three times and were mounted using an Antifade Mounting Medium with DAPI (#H‐1200, VECTOR) afterwards.

### 2.9 RNA sequencing and gene set enrichment analysis

Total RNAs of hMSC with adipogenic induction for 5 days were extracted using a RNeasy mini kit (Qiagen). Libraries were prepared using the Illumina TrueSeq mRNA sample preparation kit according to the manufacturer's instruction, and single‐end sequenced on an Illumina HiSeq 3000 machine as previously described.^26^ Reads were mapped to human genome (UCSC hg19) using STAR_2.6.0a. Differentially expressed genes and transcripts were analysed using DESeq2. Genes showing ≥1.5‐fold change (*P*‐adj ≤ .05) were considered to be significantly differentially expressed.

For gene set enrichment analysis (GSEA), we imported our gene list of interest into the GSEA software (http://www.broad.mit.edu/GSEA, v.4.0.2) and examined with the gene sets for adipogenesis pathway obtained from GSEA online database. *P* values were computed using a bootstrap distribution created by resampling gene sets of the same cardinality.

### 2.10 Chromatin immunoprecipitation assay

Chromatin immunoprecipitation (ChIP) assays were performed utilizing EZ‐Zyme™ Chromatin Prep Kit (#17‐375, Millipore) and EZ‐Magna ChIP™ HiSens Chromatin Immunoprecipitation Kit (#17‐10461, Millipore) according to the manufacturer's protocol. The antibodies used for ChIP assay were anti‐AFF1 (#A302‐344A, Bethyl, 4 μg/test) and control IgG (#CS200581, Millipore, 4 μg/test). Real‐time PCR was performed to quantify the precipitated DNA samples. Data are shown as the expression percentage of input DNA.^27^ The primers are listed in Table S1.

### 2.11 Statistics

All data are shown as mean ± SEM. Statistically significant differences were calculated by unpaired two‐tailed Student's *t* test for two groups comparison, or by one‐way ANOVA followed by Tukey's post hoc test for multiple comparisons. A *P* value < .05 was considered statistically significant.

## 3 RESULTS

### 3.1 Expression of AFF1

**FIGURE 1:**
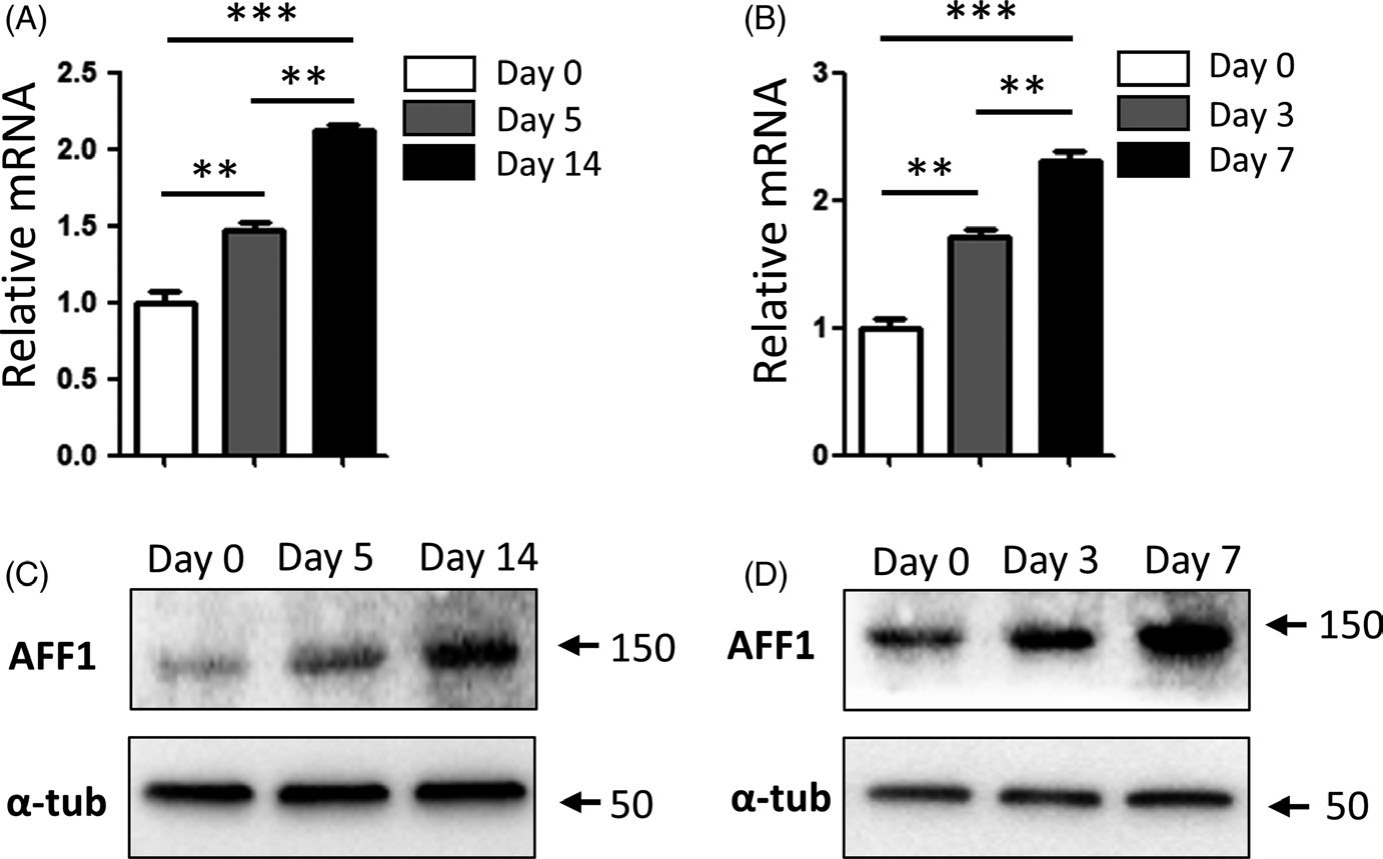
Expression of AFF1 in hMSCs and 3T3‐L1 pre‐adipocytes. A, qPCR analysis of *AFF1* expression of hMSCs during adipogenic differentiation. B, qPCR analysis of *Aff1* expression of mouse 3T3‐L1 pre‐adipocytes during adipogenic differentiation. C, Western blot analysis of AFF1 in hMSCs during adipogenic differentiation. D, Western blot analysis of AFF1 in mouse 3T3‐L1 pre‐adipocytes during adipogenic differentiation. n = 3, by one‐way ANOVA with Tukey's post hoc test. Results are shown as mean ± SEM, **P < .01 and ***P < .001

To study the potential role of AFF1 in adipogenesis, we first examined the expression of AFF1 during adipogenic induction in both hMSCs and 3T3‐L1 pre‐adipocytes. The relative mRNA levels of adipogenic‐related genes were significantly elevated in both cells at an early timepoint after induction (Figure 1A,B), and continued to increase at a later stage (Figure 1A,B). Western blot analyses confirmed that the protein levels of AFF1 were increased during adipogenic induction (Figure 1C,D).

### 3.2 Depletion of AFF1 promotes adipogenic differentiation of human MSCs

**FIGURE 2:**
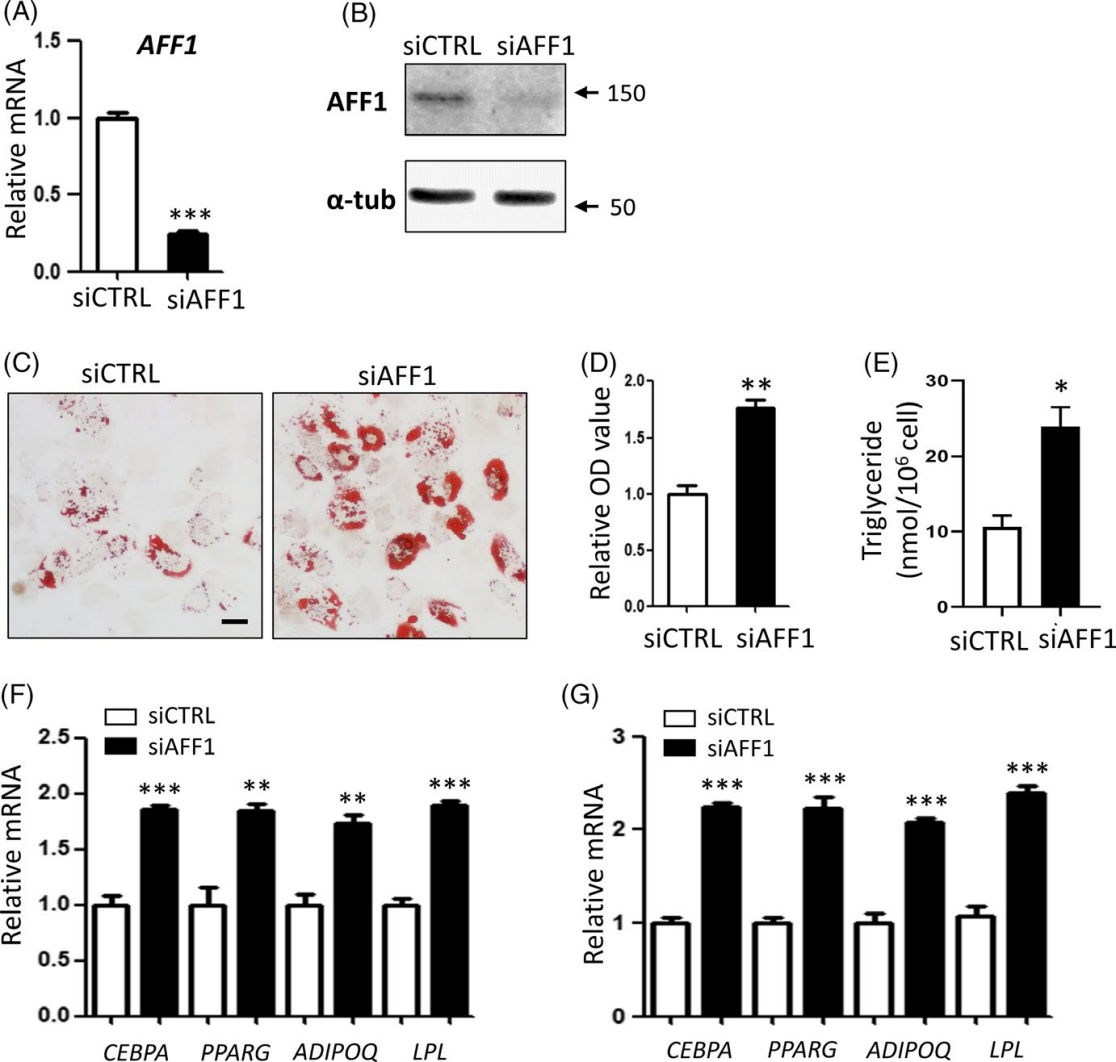
Depletion of AFF1 promotes adipogenic differentiation of human MSCs. A, qPCR shows the successful knockdown of *AFF*1. B, Western blot analysis of AFF1. C, D, Representative images and quantitative analyses of Oil Red O staining 14 d after adipogenic differentiation. Scale bar, 25 μm. E, Measurement of triglyceride (TAG) content in hMSCs 14 d after adipogenic differentiation. F, qPCR results of mRNA expressions of adipocyte‐specific molecular markers *CEBPA*, *PPARG*, *ADIPOQ* and *LPL* 7 d after differentiation. G, qPCR results of mRNA expressions of adipocyte‐specific molecular markers 14 d after differentiation. n = 3, by *t* test. Results are shown as mean ± SEM, *P < .05, **P < .01 and ***P < .001

Next, we depleted *AFF1* in hMSCs using siRNA. As shown in Figure 2A,B, qPCR and Western blot analyses confirmed a desirable knockdown efficiency. After adipogenic induction for 14 days, increased lipid accumulation was observed in *AFF1*‐depleted hMSCs, as visualized by Oil Red O staining (Figure 2C). Quantitative analyses of Oil Red O staining and triglyceride (TAG) content confirmed the elevated adipogenic potential of *AFF1*‐depleted hMSCs (Figure 2D,E). Additionally, qPCR analyses demonstrated that mRNA expressions of adipogenic‐related genes, *CEBPA*, *PPARG*, *ADIPOQ* and *LPL* were significantly up‐regulated upon AFF1 depletion (Figure 2F,G).

### 3.3 Depletion of Aff1 promotes adipogenesis of 3T3‐L1 pre‐adipocytes

**FIGURE 3:**
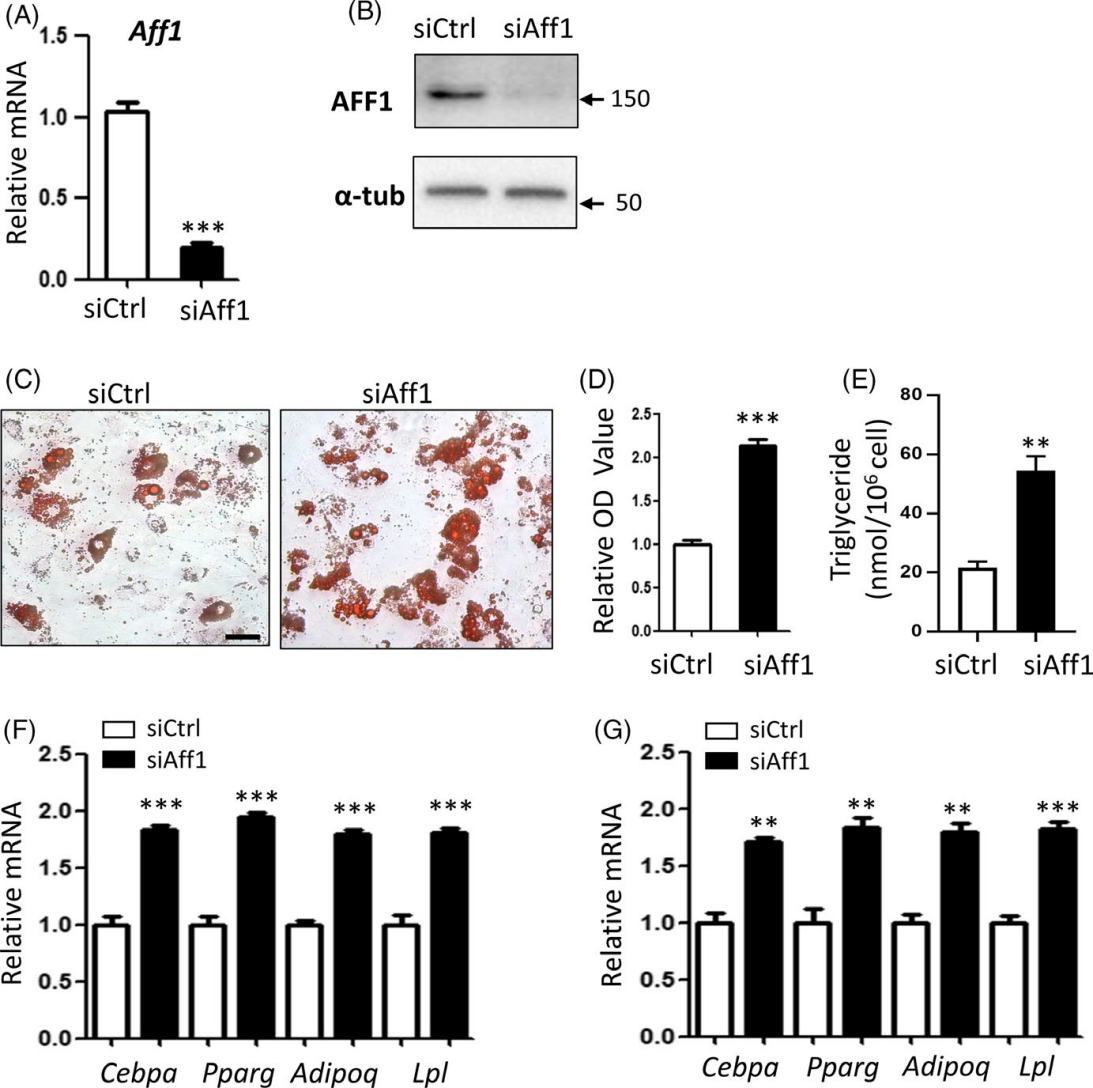
Depletion of Aff1 promotes adipogenesis of 3T3‐L1 pre‐adipocytes. A, qPCR shows the successful knockdown of *Aff1*. B, Western blot analysis of AFF1. C, D, Representative images and quantitative analyses of Oil Red O staining 7 d after adipogenic differentiation. Scale bar, 20 μm. E, Measurement of triglyceride (TAG) content in hMSCs 7 d after adipogenic differentiation. F, qPCR results of mRNA expressions of adipocyte‐specific molecular markers *Cebpa*, *Pparg*, *Adipoq* and *Lpl* 3 d after differentiation. G, qPCR results of mRNA expressions of adipocyte‐specific molecular markers 5 d after differentiation. n = 3, by *t* test. Results are shown as mean ± SEM, **P < .01 and ***P < .001

We then depleted *Aff1* in mouse 3T3‐L1 pre‐adipocytes using siRNA and confirmed the knockdown efficiency (Figure 3A,B). Likewise, *Aff1*‐depleted cells exhibited an increased lipid accumulation after 7 days of adipogenic induction, as indicated by Oil Red O staining and quantitative analysis (Figure 3C,D). An increased content of TAG was also observed (Figure 3E). Besides, expressions of adipogenic‐related genes, *Cebpa*, *Pparg*, *Adipoq* and *Lpl,* were elevated in *Aff1*‐depleted 3T3‐L1 cells (Figure 3F,G).

### 3.4 Overexpression of Aff1 impairs adipogenic differentiation

**FIGURE 4:**
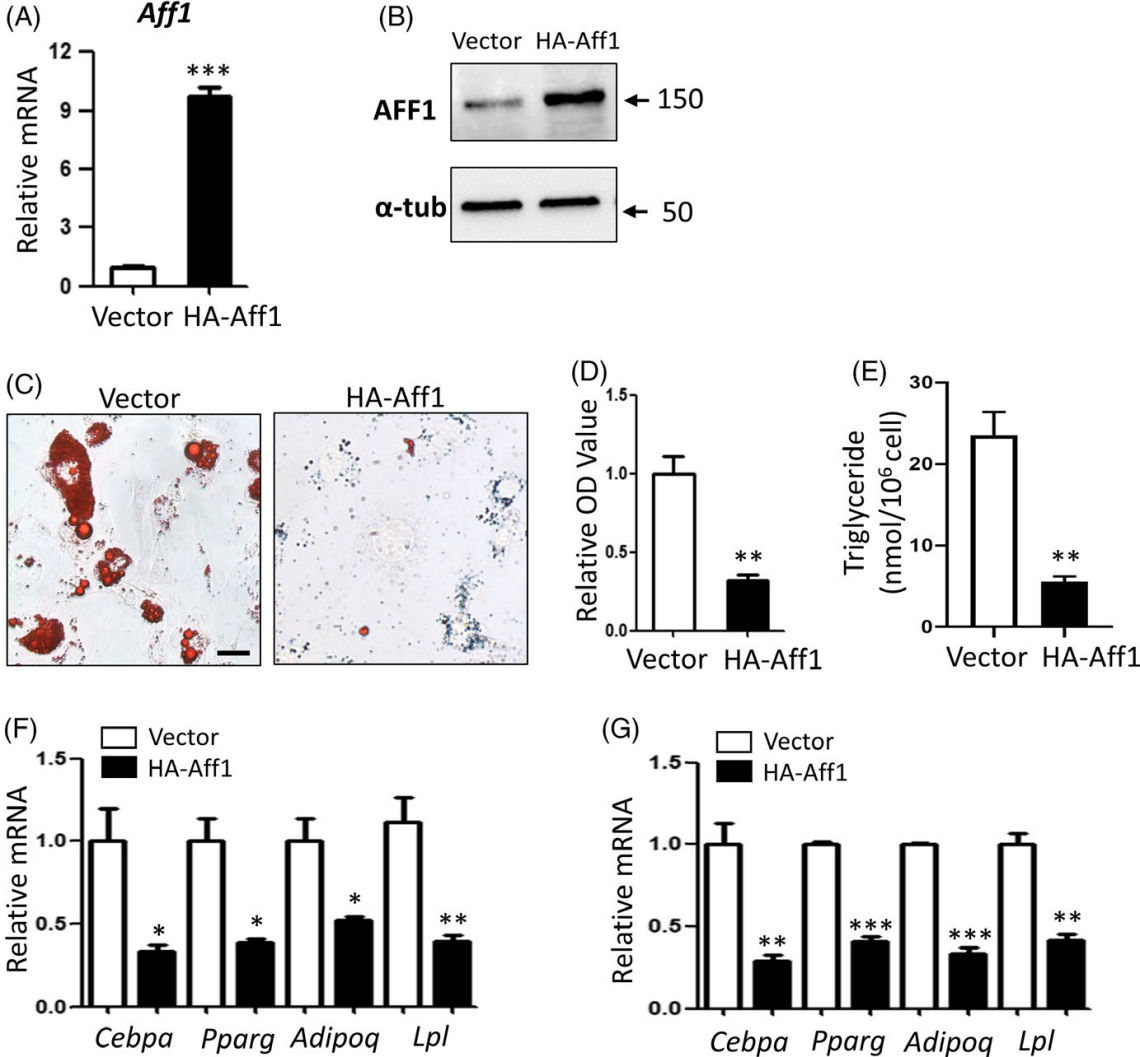
Overexpression of Aff1 impairs adipogenic differentiation in 3T3‐L1 pre‐adipocytes. A, qPCR shows the successful overexpression of *Aff1*. B, Western blot analysis of AFF1. C, D, Representative images and quantitative analyses of Oil Red O staining 7 d after adipogenic differentiation. Scale bar, 20 μm. E, Measurement of triglyceride (TAG) content in hMSCs 7 d after adipogenic differentiation. F, qPCR results of mRNA expressions of adipocyte‐specific molecular markers *Cebpa, Pparg, Adipoq* and *Lpl* 3 d after differentiation. G, qPCR results of mRNA expressions of adipocyte‐specific molecular markers 5 d after differentiation. n = 3, by *t* test. Results are shown as mean ± SEM, *P < .05, **P < .01 and ***P < .001

To verify the effect of AFF1 in adipogenic differentiation, we transduced 3T3‐L1 cells with lentiviral particles expressing HA‐Aff1. As confirmed by qPCR and Western blot, overexpression of Aff1 was successfully achieved at both mRNA and protein levels (Figure 4A,B). Notably, lipid accumulation was markedly diminished in HA‐Aff1 cells when compared with the vector group (Figure 4C). Quantification of the staining and triglyceride content validated this decline in adipogenesis of 3T3‐L1 pre‐adipocytes after Aff1 overexpression (Figure 4D,E). Next, we extracted mRNA from cells treated with vector or HA‐Aff1 after 3 or 5 days of adipogenic differentiation induction and performed qPCR analysis. Consistently, the significantly decreased mRNA levels of adipocyte‐specific molecular markers were observed in HA‐Aff1 group in comparison with the vector group (Figure 4F,G).

### 3.5 Overexpression of Aff1 inhibits adipogenesis in vivo

**FIGURE 5:**
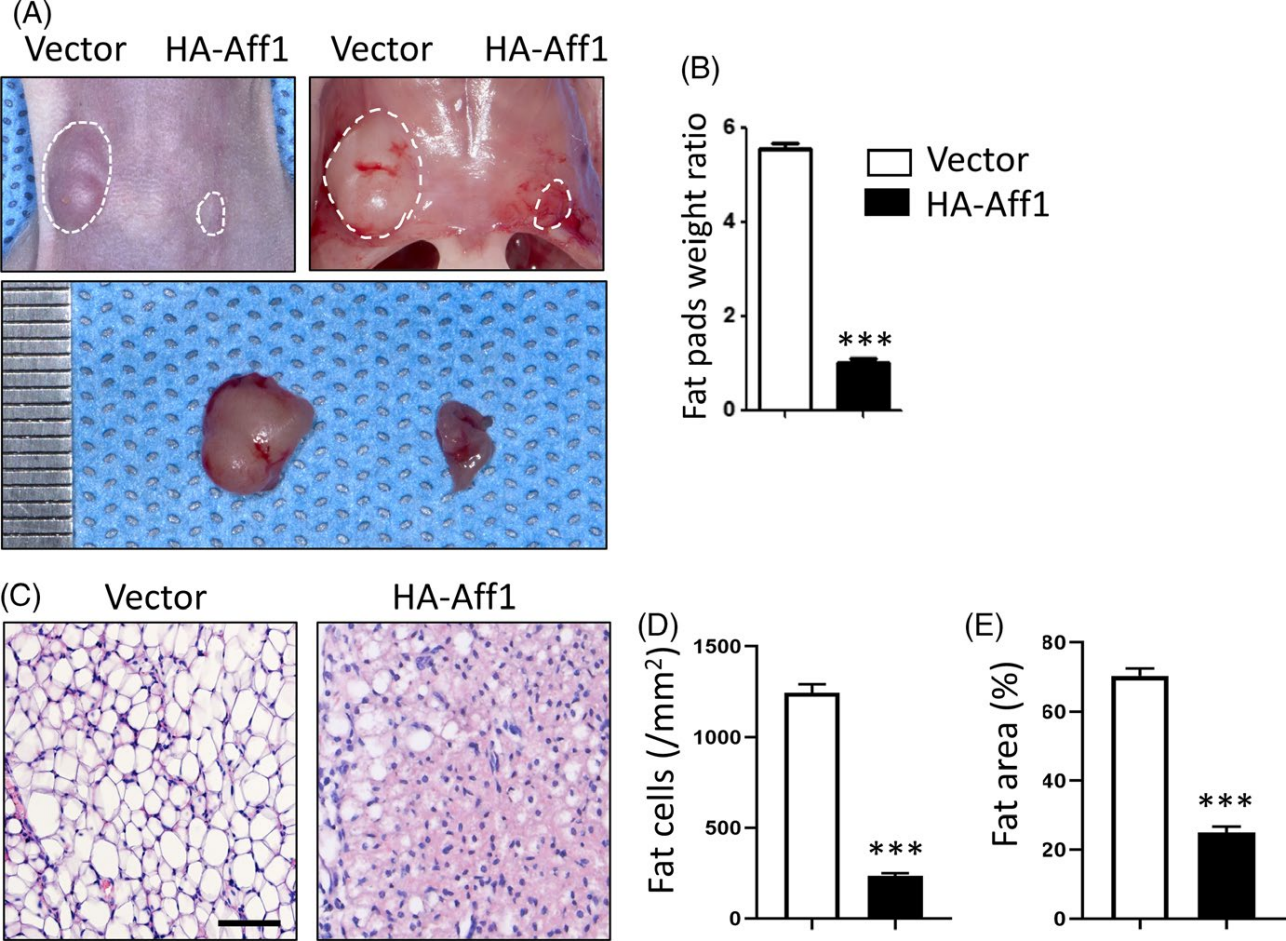
Overexpression of Aff1 inhibits adipogenesis in vivo. A, Representative image of ectopic adipose tissue formation in nude mice. Overexpression of Aff1 inhibits adipogenesis (right) compared to vector treated 3T3‐L1 cells (left). B, Quantitative analysis of fat pads weight ratio. C, H&E staining of fat pad sections. Scale bar, 50 μm. D, E, Quantitative analyses of the number of adipocytes (/mm^2^) and the percentage of fat area versus total tissue area (%). n = 6, by *t* test. Results are shown as mean ± SEM, ***P < .001

Next, we sought to investigate the effect of AFF1 towards adipogenesis in vivo via an ectopic adipose formation model. 3T3‐L1 pre‐adipocytes stably expressing vector or HA‐Aff1 were bilaterally injected into the different sides of the sternal area of nude mice. After 6 weeks, fat pads were much smaller and weighed less at the HA‐Aff1 side in comparison with the vector controls (Figure 5A,B). Histological analyses also showed that the number and area of fat cells within the engrafted tissues were reduced in the HA‐Aff1 group (Figure 5C‐E).

### 3.6 AFF1 regulates *Tgm2* transcription

**FIGURE 6:**
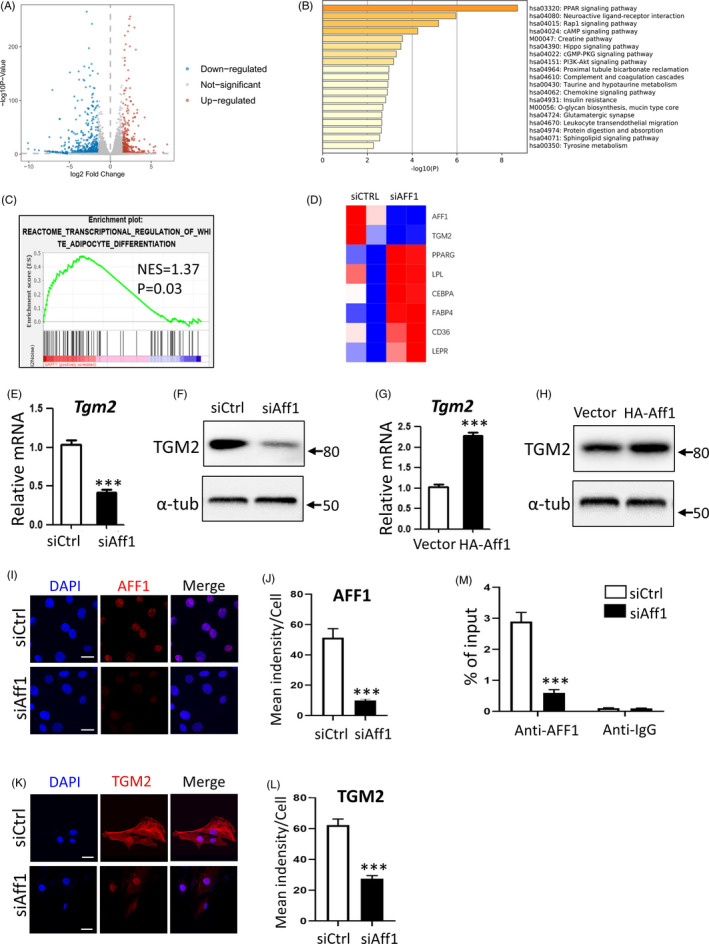
AFF1 regulates *TGM2* transcription. A, Volcano plot of RNA‐seq analysis. A total of 616 genes are up‐regulated and 361 genes are down‐regulated in *AFF1*‐depleted hMSCs. B, KEGG pathway analysis. C, GSEA shows an increased enrichment of adipogenic differentiation‐related genes in *AFF1*‐depleted hMSCs. D, Heatmap of representative genes associated with adipogenic differentiation. E, qPCR shows a decreased expression of *Tgm2* in *Aff1*‐depleted 3T3‐L1 cells. F, Western blot analysis of TGM2 in *Aff1*‐depleted 3T3‐L1 cells. G, qPCR shows an enhanced expression of *Tgm2* in HA‐Aff1 3T3‐L1 pre‐adipocytes. H, Western blot analysis of TGM2 in HA‐Aff1 overexpressed 3T3‐L1 pre‐adipocytes. I, J, Immunofluorescence staining of AFF1 and quantification. Scale bar, 20 μm. K, L, Immunofluorescence images and quantitative analysis show a decreased TGM2 expression in *Aff1*‐depleted 3T3‐L1 pre‐adipocytes. Scale bar, 20 μm. M, ChIP assay for AFF1 shows that it binds to the promoter region of *Tgm2*. n = 3, by *t* test. Results are shown as mean ± SEM, ***P < .001

To elucidate the mechanism, we performed RNA‐seq, and found a total of 616 up‐regulated genes and 361 down‐regulated genes in *AFF1*‐deficient hMSCs (Figure 6A). KEGG pathway analysis revealed that AFF1 was related with multiple signalling pathways, such as PPARG, Rap1, Hippo, and PI3K‐Akt (Figure 6B). Gene set enrichment analysis confirmed the enhanced adipogenic differentiation after AFF1 depletion (Figure 6C). The adipogenic markers, such as PPARG, CEBPA, LPL, FABP4, CD36 and LEPR, were significantly up‐regulated (Figure 6D).

Notably, RNA‐seq data also unveiled that the transcription of Transglutaminase 2 (TGM2), a pivotal inhibitor of adipogenesis,^28^ was largely decreased (*P* < .001) (Figure 6D). To validate this observation, we then performed qPCR and Western blot analyses. The expression of Tgm2 was significantly decreased in *Aff1*‐depleted 3T3‐L1 cells (Figure 6E,F), while Aff1 overexpression markedly elevated its expression (Figure 6G,H). In addition, immunofluorescence staining result showed a significant decrease of TGM2 in *Aff1*‐depleted cells in comparison with controls (Figure 6I‐L).

Next, we performed ChIP‐qPCR assay on the promoter region of *Tgm2*. Notably, the enrichment of AFF1 at the promoter of *Tgm2* was significantly decreased in response to the depletion of Aff1 (Figure 6M), indicating that AFF1 directly regulates *Tgm2* transcription.

### 3.7 Overexpression of Tgm2 partially rescues adipogenic differentiation of *Aff1*‐depleted cells

**FIGURE 7:**
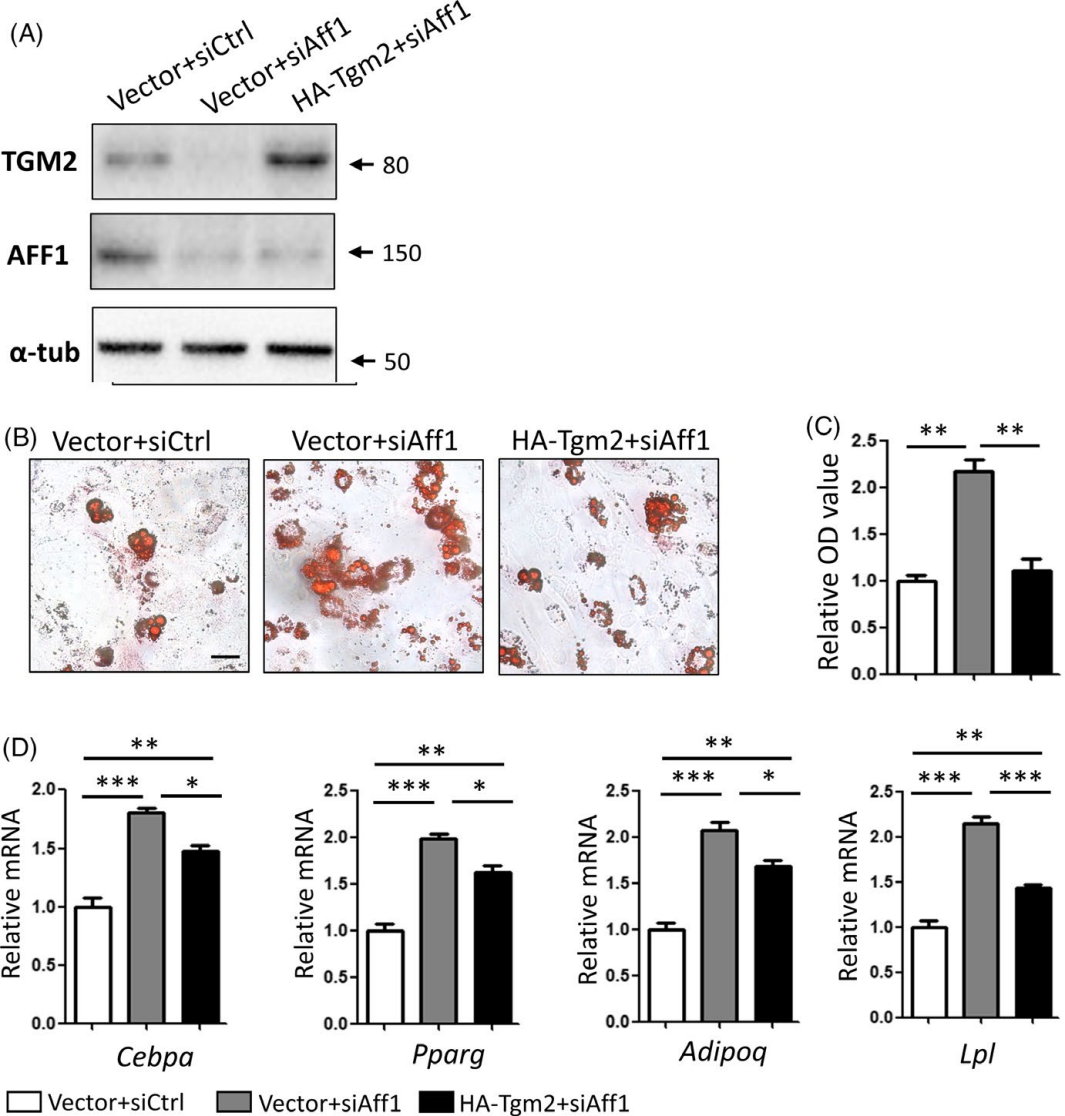
Overexpression of Tgm2 partially rescues adipogenic differentiation of *Aff1*‐depleted cells. A, Western blot analyses of TGM2 and AFF1. B, C, Representative images and quantitative analyses of Oil Red O staining 7 d after adipogenic differentiation. Scale bar, 20 μm. D, qPCR results of adipocyte‐specific molecular markers *Cebpa, Pparg, Adipoq* and *Lpl* after adipogenic induction for 3 d. n = 3, by one‐way ANOVA with Tukey's post hoc test. Results are shown as mean ± SEM, *P < .05, **P < .01 and ***P < .001

Next, we carried out rescue experiments by overexpressing Tgm2 using lentivirus particles. Overexpression of Tgm2 successfully restored the protein level of TGM2 in Aff1*‐*deficient cells (Figure 7A) and attenuated the augmented lipid accumulation caused by Aff1 depletion, as indicated by Oil Red O staining and quantitative analyses (Figure 7B,C). Besides, the mRNA expressions of adipogenic‐related genes were largely maintained at 3 days after adipogenic induction (Figure 7D).

## 4 DISCUSSION

Transcriptional and epigenetic regulation plays an important role in adipogenesis.^29^ In the present work, we delineated AFF1 as a previously unknown inhibitor of adipogenic differentiation. Our present data show that depletion of AFF1 in hMSCs and pre‐adipocytes enhances adipogenesis, while AFF1 overexpression results in reduced ectopic fat tissue formation in vivo. In addition, we find that AFF1 directly regulates the transcription of TGM2.

Super elongation complex has been elucidated as a main regulator in gene transcription elongation.^16^ AFF1 constitutes the scaffold of SEC and acts as the bridge stabilizing and connecting other subunits.^30^ As the main functional protein in SEC, P‐TEFb could phosphorylate RNA Pol II C‐terminal domain (CTD), leading to the dissociation of paused Pol II from DRB sensitivity‐inducing factor (DSIF) and negative transcription elongation factor (NELF) for productive transcriptional elongation.^31^ AFF1 is necessary to recruit P‐TEFb to steer the transcriptional elongation control and shows a crucial function in regulating gene transcription.^32^ The missense mutation of murine *Aff1* could cause a “robotic” phenotype which manifested as neurodegeneration, suggesting AFF1 may play an important role in regulating neural stem cell.^33^ There has been no data indicating the specific adipogenic phenotype of AFF1 in transgenic mice so far. Besides, the function of AFF1 in cellular adipogenesis is still unreported. Accordingly, in the current study, we show that the deletion of AFF1 in hMSCs significantly increases the expression of *CEBPA, PPARG, ADIPOQ* and other adipogenic genes by performing RNA‐seq, which specifies that AFF1 takes an important part in transcriptional cascade regulation of adipogenesis. This discovery might lay a solid foundation for further adipogenic phenotype study on AFF1 in the near future.

TGM2 is widely expressed in body tissues such as fat, bone, cartilage, kidney, liver, heart, lung and nervous tissue.^34^, ^35^, ^36^ The biological function of TGM2 has been broadly implicated in various aspects including cell differentiation and maturation, cell morphology and adhesion, cell death, ECM stabilization and cell adipogenesis.^28^, ^34^, ^37^ Previous work has proved that TGM2 and factor XIII‐A (FXIII‐A), another member of transglutaminase enzyme family, are both identified in white adipose tissue.^38^ Recently, TGM2 has been identified as a novel adipogenesis inhibitor.^28^ *Tgm2*‐deficient mouse embryonic fibroblasts (MEFs) displayed an increased and accelerated lipid accumulation. Increased expression of major adipogenic transcription factors, PPARγ and C/EBPα were detected in *Tgm2*‐deficient MEFs.^28^

Here, we show that AFF1 can bind to the promotor of *TGM2* and regulate its transcription. Previous studies showed that TGM2 expression is regulated by CDK9, which holds core function in composing P‐TEFb complex, indicating TGM2 as P‐TEFb dependent.^39^ In addition, an enhanced occupancy of P‐TEFb at TGM‐2 loci near the 30 end of the ORFs was also revealed by chromatin immunoprecipitation.^40^ As AFF1 facilitates the transcription elongation function of P‐TEFb, we suppose that AFF1 up‐regulates TGM2 transcription by recruiting P‐TEFb to its promotor, which needs further investigation.

Previously, we verified that AFF1 inhibited osteogenic differentiation of hMSCs by directly targeting Wnt signalling antagonist DKK1.^21^ Theoretically, the osteogenic and adipogenic differentiation of MSCs present an inverse correlation and these two phenotypes often take place at the expense of the other.^41^ Many signalling pathways, including Wnt/β‐catenin signalling, play opposite roles in balancing osteogenic and adipogenic differentiation of MSCs.^42^, ^43^ In this study, however, we did not detect a significant variation of Wnt signalling pathway by RNA‐seq analysis. Collectively, our data suggest that AFF1 may regulate different biological processes through distinct pathways.

In summary, we demonstrate that AFF1 inhibits adipogenesis both in human MSCs and mouse 3T3‐L1 pre‐adipocytes. Mechanically, AFF1 regulates the transcription of a novel adipogenesis inhibitor TGM2. Our data indicate AFF1 as a previously unknown regulator of adipogenesis.

## CONFLICT OF INTEREST

The authors declare that there is no conflict of interest regarding the publication of this paper.

## AUTHOR CONTRIBUTION

YC, YW and QY designed the project; YC, YW, RS, RX and CZ performed the experiments; YC, YW, LW, RS and Yunshu Wu analysed the data; YC, YW and QY wrote and edited the manuscript. All authors reviewed the manuscript.

## Supporting information

Table S1

## Data Availability

The authors declare that all data that support the findings of this study are available from the corresponding author upon reasonable request.
