# Supplementary material for: AFF1 inhibits adipogenic differentiation via targeting TGM2 transcription
Source: Cell Prolif. 2020 May 22;53(6):e12831. doi: 10.1111/cpr.12831 (PMC7309944; doi:10.1111/cpr.12831)
Supplement: Supplementary file 1 — Table S1 [file CPR-53-e12831-s001.docx]

Supplementary table 1. Primers for qPCR and ChIP-PCR.

| **qRT-PCR (human)** | |  | |
| --- | --- | --- | --- |
| **Genes** | **Primers** | **Sequences (5*’*‐3*’*)** | |
| ***36B4*** | Forward | AGCCCAGAACACTGGTCTC | |
|  | Reverse | ACTCAGGATTTCAATGGTGCC | |
| ***AFF1*** | Forward | TACAATGACGACAGAAACCTGC | |
|  | Reverse | GGCGATGAGTGTGAGACTTAGTA | |
| ***ADIPOQ*** | Forward | CCCTCTCTTACAAGCCCATCA | |
|  | Reverse | GAGCCAGTCTGGTAGTACATCA | |
| ***PPARG*** | Forward | ACCAAAGTGCAATCAAAGTGGA | |
|  | Reverse | ATGAGGGAGTTGGAAGGCTCT | |
| ***CEBPA*** | Forward | TTCACATTGCACAAGGCACT | |
|  | Reverse | GAGGGACCGGAGTTATGACA | |
| ***LPL*** | Forward | TCATTCCCGGAGTAGCAGAGT | |
|  | Reverse | GGCCACAAGTTTTGGCACC | |
| **qRT-PCR (mouse)** | | |  |
| **Genes** | **Primers** | | **Sequences (5*’*‐3*’*)** |
| ***36B4*** | Forward | | TGAGATTCGGGATATGCTGTTGG |
|  | Reverse | | CGGGTCCTAGACCAGTGTTCT |
| ***Aff1*** | Forward | | GAAGGAAAGACGCAACCAAGA |
|  | Reverse | | TAGCTCATCGCCTTTTGCAGT |
| ***Tgm2*** | Forward | | GACAATGTGGAGGAGGGATCT |
|  | Reverse | | CTCTAGGCTGAGACGGTACAG |
| ***Adipoq*** | Forward | | CGTCACTGTTCCCAATGT |
|  | Reverse | | ACCGTGATGTGGTAAGAG |
| ***Pparg*** | Forward | | CATCAGGCTTCCACTATG |
|  | Reverse | | CACAGCAAGGCACTTCTG |
| ***Cebpa*** | Forward | | ACTCCTCCTTTTCCTACCG |
|  | Reverse | | AGGAAGCAGGAATCCTCC |
| ***Lpl*** | Forward | | GGGAGTTTGGCTCCAGAGTTT |
|  | Reverse | | TGTGTCTTCAGGGGTCCTTAG |
| **ChIP-PCR (mouse)** | | |  |
| **Genes** | **Primers** | | **Sequences (5*’*‐3*’*)** |
| ***Tgm2*** | Forward | | CTTGTCCTTTGGGAATGTCTGA |
|  | Reverse | | GGAGTTTGTGGGTAGAGCCTTG |
